# Rationally-defined microbial consortia suppress multidrug-resistant proinflammatory Enterobacteriaceae via ecological control

**DOI:** 10.21203/rs.3.rs-3462622/v1

**Published:** 2023-10-23

**Authors:** Kenya Honda, Munehiro Furuichi, Takaaki Kawaguchi, Marie-Madlen Pust, Keiko Yasuma-Mitobe, Damian Plichta, Naomi Hasegawa, Takashi Ohya, Shakti Bhattarai, Satoshi Sasajima, Aoto Yoshimasa, Timur Tuganbaev, Mizuki Yaginuma, Masahiro Ueda, Nobuyuki Okahashi, Kimiko Amafuji, Yuuko Kiridooshi, Kayoko Sugita, Martin Stražar, Ashwin Skelly, Wataru Suda, Masahira Hattori, Nobuhiro Nakamoto, Silvia Caballero, Jason Norman, Bernat Olle, Takeshi Tanoue, Makoto Arita, Vanni Bucci, Koji Atarashi, Ramnik Xavier

**Affiliations:** Keio University School of Medicine; Keio University School of Medicine; Keio University School of Medicine; Broad Institute of MIT and Harvard; Keio University School of Medicine; Broad Institute; Keio University School of Medicine; Keio University School of Medicine; UMass Chan Medical School; Keio University School of Medicine; JSR-Keio University Medical and Chemical Innovation Center; Keio University School of Medicine; Keio University School of Medicine; Osaka Universtiy; Keio University School of Medicine; JSR Corp; Keio University School of Medicine; Broad Institute of MIT and Harvard; Keio University School of Medicine; RIKEN Center for Integrative Medical Sciences; RIKEN IMS; Keio University School of Medicine; Immunology Program, Sloan Kettering Institute, Memorial Sloan Kettering Cancer Center; Vedanta Biosciences Inc.; Vedanta Biosciences; Keio University School of Medicine; RIKEN Center for Integrative Medical Sciences; UMass Chan Medical School; Keio University School of Medicine; Massachusetts General Hospital

## Abstract

Persistent colonization and outgrowth of pathogenic organisms in the intestine may occur due to long-term antibiotic usage or inflammatory conditions, which perpetuate dysregulated immunity and tissue damage^1,2^. Gram-negative *Enterobacteriaceae* gut pathobionts are particularly recalcitrant to conventional antibiotic treatment^3,4^, though an emerging body of evidence suggests that manipulation of the commensal microbiota may be a practical alternative therapeutic strategy^5–7^. In this study, we rationally isolated and down-selected commensal bacterial consortia from healthy human stool samples capable of strongly and specifically suppressing intestinal *Enterobacteriaceae*. One of the elaborated consortia, consisting of 18 commensal strains, effectively controlled ecological niches by regulating gluconate availability, thereby reestablishing colonization resistance and alleviating antibiotic-resistant *Klebsiella*-driven intestinal inflammation in mice. Harnessing these microbial activities in the form of live bacterial therapeutics may represent a promising solution to combat the growing threat of proinflammatory, antimicrobial-resistant bacterial infection.

The discovery and clinical application of potent antimicrobial agents has been a double-edged sword, saving countless lives worldwide while simultaneously spurring the evolution and expansion of multidrug-resistant organisms (MDROs) that have become an eminent threat to global health. In particular, Gram-negative *Enterobacteriaceae* including *Escherichia* and *Klebsiella* species have emerged globally as multidrug-resistant nosocomial pathogens for which there are limited therapeutic options^3,4^. In addition to antibiotic treatments, inflammatory conditions also predispose to *Enterobacteriaceae* outgrowth^1,2,8–13^. Indeed, chronic inflammatory intestinal conditions such as inflammatory bowel disease (IBD) are often associated with dysbiosis and enrichment of *Enterobacteriaceae*^14,15^. An emerging body of evidence suggests that persistence of Enterobacteriaceae helps perpetuate intestinal inflammation and nosocominal infections of other microbes^16–19^. Furthermore, an increased relative abundance of *Enterobacteriaceae* in the gut is associated with higher mortality risk in the general population^20^. Several clinical and preclinical studies have found faecal microbiota transplantation (FMT) to be efficacious in reducing levels of drug-resistant and proinflammatory *Enterobacteriaceae* in the intestine^5–7^. Therefore, manipulation of the microbiota represents a promising approach to treat MDRO infection and IBD. However, FMT therapeutics are plagued by mixed results, safety concerns, and production impracticalities secondary to inherent batch-to-batch variability^5^. Overcoming these limitations requires the identification of defined microbial consortia that can be manufactured at large-scale and obviating the need for human donors. To this end, it is imperative to delineate specific bacteria or consortia capable of decolonizing *Enterobacteriaceae* and elucidate their mechanisms of action.

## Isolation of an 18-strain consortium capable of efficiently decolonizing *Klebsiella*

**Figure 1:**
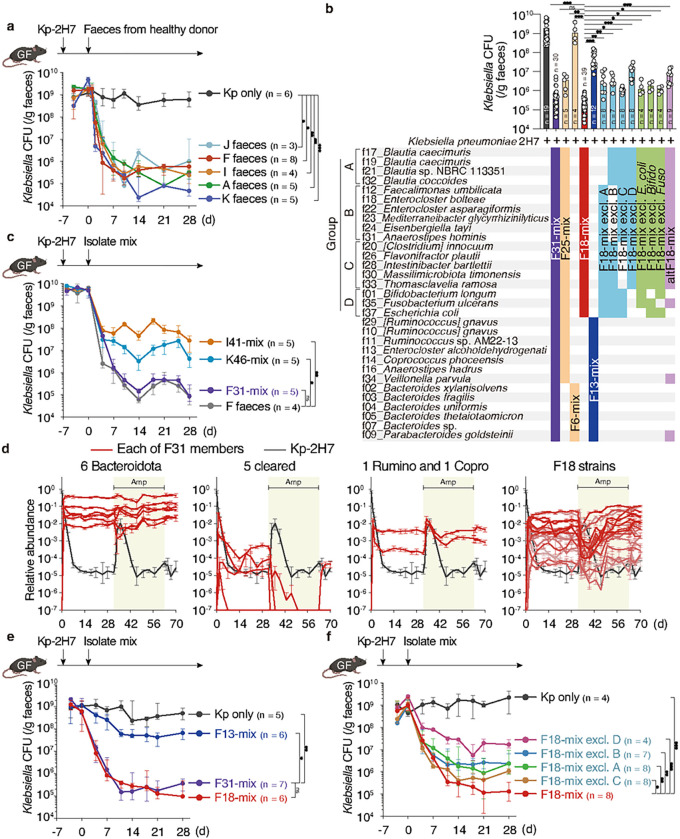
Elaboration of an 18 strain-consortium capable of decolonizing *Klebsiella*. **a-c, e, f,** Germ-free (GF) C57BL/6 mice were monocolonized with Kp-2H7, followed by oral administration of stool samples from one of five healthy human donors (A, F, I, J, and K) (**a**) or the indicated mixture of bacterial isolates (**b, c, e, f**). Faecal CFU of Kp-2H7 throughout the experiment (**a, c, e, f**). The combination of strains administered and faecal CFU of Kp-2H7 on day 28 are shown in **b**. **d**, GF mice (n = 5) were monocolonized with Kp-2H7, followed by oral administration of F31-mix. Ampicillin (200 mg/L) was given in the drinking water from day 32 to 63. Relative abundance of each of the 31 strains was quantified by qPCR in two technical replicates and average data are shown. In **a-c**, **e**, and **f**, median ± IQR (interquartile range) are shown for representative data from two independent experiments with similar results. *P < 0.05, **P < 0.01, ***P < 0.001; ns, not significant; by Kruskal-Wallis test using the Benjamini-Hochberg correction for multiple comparisons at day 28.

*Klebsiella* species comprise a major etiology of nosocomial infections^3,4^. We previously isolated multidrug-resistant *Klebsiella* strains from IBD patients, including the *K. pneumoniae* Kp-2H7 strain, which can expand and persist in the intestine in the setting of antibiotic-induced dysbiosis and promote T helper 1 (T_H_1) cell-mediated inflammation^17^. We set out to identify human gut commensals that promote decolonization of Kp-2H7 using the strategy outlined in **Extended Data Fig. 1a**. Germ-free (GF) mice were monocolonized with Kp-2H7 and orally inoculated with a stool sample from one of five healthy Japanese donors (A, F, I, J, or K) 7 days later. Efficacy of Kp-2H7 decolonization by FMT from each donor was examined by longitudinally quantifying faecal Kp-2H7 abundance. FMT from all donors resulted in a 3–4 log reduction in Kp-2H7 abundance (Fig. 1a). We selected stool samples from donors F, I, and K for follow-up analysis, and cultured them using six different types of media. We isolated 37 strains (31 unique strains when deduplicated) from donor F, 41 strains from donor I, and 46 strains from donor K based on 16S rRNA gene sequencing followed by whole-genome sequence analysis (**Table S1**). Isolate amplicon sequence variants (ASVs) accounted for 82%, 60%, and 48% of total microbiota sequences from donors F, I, and K, respectively (**Extended Data Fig. 1b**). A mixture of bacterial strains isolated from each donor was inoculated into Kp-2H7-monocolonized mice and *Klebsiella* decolonization capacity was queried. The mixture of 31 strains from donor F (designated as F31-mix) was most effective, and the magnitude and kinetics of Kp-2H7 reduction were comparable to those induced by donor F FMT (Fig. 1b, c).

To identify a minimal effector consortium from F31-mix, mice monocolonized with Kp-2H7 (day −7) were treated with F31-mix (day 0) and then given ampicillin via the drinking water (day 32 to 63) to perturb microbiota homeostasis (Fig. 1d and **Extended Data Fig. 2a**). Of note, Kp-2H7 carries b-lactamase genes and is resistant to ampicillin^17^. The faecal abundance of each of the 31 strains was longitudinally quantified by qPCR. Ampicillin treatment resulted in a transient surge in Kp-2H7 abundance, whereas the 31 strains showed variable trajectories (Fig. 1d and **Extended Data Fig. 2a**). The majority of Bacillota (formerly Firmicutes) strains exhibited an inverse abundance pattern compared to Kp-2H7, whereas Bacteroidota strains remained largely unchanged (Fig. 1d and **Extended Data Fig. 2a**). These results suggested that the Bacteroidota strains might not be contributing to Kp-2H7 suppression. Therefore, we divided the F31 strains into two groups: six Bacteroidota strains (F6-mix) and 25 other strains (F25-mix). F25-mix reduced Kp-2H7 abundance to a similar extent as did F31-mix, whereas F6-mix had no effect (Fig. 1b and **Extended Data Fig. 3a**). To further hone in on the effector strains among F25-mix, we excluded 5 strains (f34, f29, f13, f16, f11) that failed to colonize or were cleared following ampicillin treatment. In addition, we excluded a *Coprococcus* and a *Ruminococcus* strain (f14 and f10, respectively) that exhibited similar trajectories to Kp-2H7 (Fig. 1d and **Extended Data Fig. 2a**). A Spearman’s rank correlation test indicated that most of the remaining 18 strains were significantly inversely associated with Kp-2H7 abundance (**Extended Data Fig. 2b**). We thus tested the activity of these 18 strains together (F18-mix) and observed robust Kp-2H7 decolonization, with similar magnitude and kinetics to mice treated with the parental F31-mix (Fig. 1b and 1e). In contrast, administration of the remaining 13 strains (F13-mix) that were excluded from F18-mix was far less effective (Fig. 1b and 1e). In an attempt to further narrow down the minimal effective consortia, we next generated seven derivatives of F18-mix by subtracting various combinations of bacterial species, which ranged in size from 12 to 17 strains. These derivative consortia exhibited varying capacities to decolonize Kp-2H7 *in vivo*, though none was as effective as the full F18-mix (Fig. 1b, Fig. 1f, and **Extended Data Fig. 3b**). Specifically, F18-mix was subdivided into four phylogenetic groups and derivative subsets lacking either group A (4 *Blautia* strains), B (6 *Lachnospiraceae* strains), C (5 Bacillota strains), or D (3 strains from other phyla) were tested for their ability to decolonize Kp-2H7. Compared to the full F18-mix, all derivative subsets exhibited reduced decolonization capacity (Fig. 1b and Fig. 1f). As subtraction of group D strains showed the greatest effect, we also tested three different 17-strain mixtures, each excluding one of the three group D strains (f37_*Escherichia coli*, f35_*Fusobacterium ulcerans*, or f01_*Bifidobacterium longum*) from F18-mix. These three 17-mixes demonstrated a significantly reduced ability to decolonize Kp-2H7 as compared to the full F18-mix (Fig. 1b and **Extended Data Fig. 3b**). An alternate F18-mix (altF18-mix) generated by replacing f37_*E. coli* and f01_*B. longum* with F13-mix-derived f34_*Veillonella* and f09_*Parabacteroides* also exhibited significantly reduced activity (Fig. 1b), indicating that the observed suppressive effect is not merely a function of strain number but rather depends on consortium composition. Together, these results suggest that the F18 members act cooperatively and that all phylogenetic components are required to achieve maximal Kp-2H7 suppression.

## F18-mix preferentially suppresses *Enterobacteriaceae* growth

**Figure 2:**
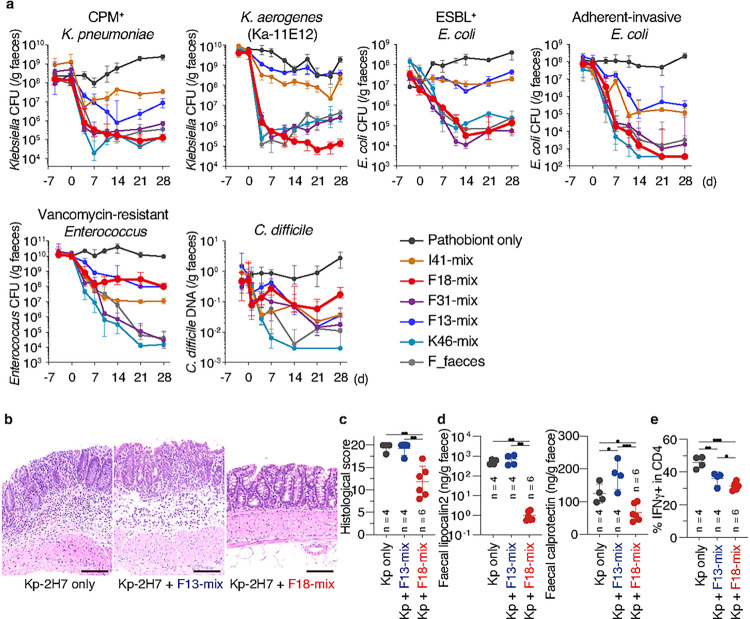
Control of intestinal pathogens and colitis by F18-mix. **a**, GF mice (n = 3–10 per group) were monocolonized with the indicated pathogenic or antibiotic-resistant (pathobiont) strain, and then treated with the indicated bacterial mixture. Faecal pathobiont load was examined by counting CFUs or by qPCR of bacterial DNA (for *C. difficile*). **b-e**, GF *Il10*^−/−^ mice were monocolonized with Kp-2H7, followed by oral administration with the indicated bacterial mix. Representative haematoxylin and eosin staining of the colon (**b**), histological colitis scores (**c**), faecal lipocalin-2 and calprotectin levels (**d**), and frequencies of IFN-γ^+^ cells among colonic lamina propria CD4^+^TCRβ^+^ T-cells (**e**) are shown. In **a** and **c-e**, median ± IQR are shown for representative data from two independent experiments with similar results. *P < 0.05, **P < 0.01, ***P < 0.001; ns, not significant; by Kruskal-Wallis test using the Benjamini and Hochberg correction for multiple comparisons (**c-e**).

The increasing incidence of extended-spectrum b-lactamase (ESBL)- and carbapenemase (CPM)-producing *Enterobacteriaceae* species has become a serious problem worldwide^4,21^. Therefore, we next examined the capacity of F18-mix to decolonize ESBL^+^ *E. coli* (ATCC BAA2777) and CPM^+^ *K. pneumoniae* (ATCC BAA1705) strains in a gnotobiotic setting. For comparison, we additionally analysed F31-, F13-, I41-, and K46-mixes, as well as donor F complete faecal microbiota. F18-mix was highly effective at suppressing ESBL^+^ *E. coli* and CPM^+^ *K. pneumonia* in the intestine, with similar magnitude and kinetics as donor F faecal microbiota, achieving 3–4 log reductions (Fig. 2a). F18-mix was also highly effective at decolonizing *Klebsiella aerogenes* (strain Ka-11E12) and adherent-invasive *E. coli* (AIEC, strain LF82), both of which have been implicated in IBD pathogenesis^17,22^ (Fig. 2a). K46-mix was as efficacious as F18-mix at decolonizing the tested *Klebsiella* and *E*. *coli* strains, whereas F13-mix and I41-mix were far less effective (Fig. 2a). Notably, members of Pseudomonadota (formerly Proteobacteria) other than Enterobacteriaceae, including *Campylobacter upsaliensis* and *Pseudomonas aeruginosa*, were largely resistant to decolonization by all tested consortia including F18-mix (**Extended Data Fig. 4a**).

We additionally investigated the effects of donor F-, K-, and I-derived consortia on gram-positive pathogens, including vancomycin-resistant *Enterococcus faecium* (VRE) and *Clostridioides difficile*, which are also listed as high-priority multidrug-resistant threats^4^. K46-mix was highly effective against both VRE (ATCC 700221) and *C. difficile* (ATCC BAA1382), whereas F18-, F13-, and I41-mixes were not (Fig. 2a). Interestingly, F31-mix (which comprises F18- and F13-mixes) was as efficacious as K46-mix at decolonizing VRE, though this effect was blunted against *C. difficile* (Fig. 2a). Collectively, these results indicate that the process of narrowing down F31-mix to F18-mix led to the selection of commensals preferentially able to decolonize *Enterobacteriaceae*.

Kp-2H7 is a strong inducer of intestinal T_H_1 cells and can act as a colitogenic pathobiont in the context of a genetically susceptible host, such as interleukin-10-deficient (*Il10*^−/−^) mice^17^. Therefore, we examined whether F18-mix-mediated Kp-2H7 decolonization can prevent the induction of colitis. GF *Il10*^−/−^ mice were monocolonized with Kp-2H7 and either F18-mix or F13-mix was orally administered 7 days later. Similarly to wild-type mice, *Il10*^−/−^ mice treated with F18-mix but not F13-mix showed a 3–4 log reduction in Kp-2H7 abundance (**Extended Data Fig. 4b**). In contrast to F13-mix, F18-mix significantly reduced histological scores of colitis, levels of faecal lipocalin-2 and calprotectin (which serve as sensitive biomarkers of intestinal inflammation), and T_H_1 cell frequency (Fig. 2b–e). Therefore, F18-mix is capable of reducing *Klebsiella* burden and alleviating IBD-like inflammation.

## Previously uncharacterized mechanisms involved in *Klebsiella* decolonization

Next, we sought to elucidate the mechanisms underlying F18-mix-mediated *Klebsiella* suppression. Since Kp-2H7 is known to induce interferon-g (IFNg)-expressing T cells in the intestine^17^, we reasoned that F18-mix might be controlling *Klebsiella* colonization via regulation of the IFNg response. However, the capacity of F18-mix to decolonize Kp-2H7 was not affected in IFNg receptor-deficient (*Ifngr1*^−/−^) mice (**Extended Data Fig. 5a**). Similarly, F18-mix was able to efficiently reduce intestinal Kp-2H7 burden in mice deficient in Toll-like receptor signaling (*Myd88*^*−/−*^*Trif*^*−/−*^) or lymphocytes (*Rag2*^−/−^*gc*^−/−^) (**Extended Data Fig. 5b**), implicating a mechanism independent of the canonical innate and adaptive immune systems. We also assessed intestinal epithelial cell transcriptomic responses in GF mice colonized with effective (F31- or F18-mix) versus less-effective (F13-mix) microbial consortia. However, we could not discern consistent shifts in genes or pathways that were specifically modulated by the effective consortia, including those encoding previously-reported putative anti-*Klebsiella* gene products like antimicrobial peptides and PPARg-regulated molecules^23–25^ (**Extended Data Fig. 5c**). Therefore, while host-intrinsic factors could still play a role in F18-mix-mediated Kp-2H7 decolonization, we decided to focus our line of inquiry on interbacterial interactions.

Caecal suspensions from mice colonized with F31- or F18-mix, but not F13-mix, strongly suppressed the growth of Kp-2H7 upon *in vitro* anaerobic coculture (**Extended Data Fig. 6a**). This suppressive effect was abrogated when coculture was performed under aerobic conditions or when the caecal suspension was filtered or heat-inactivated, suggesting that live F18-mix activity is required. We next searched for effector consortia-derived molecules that may have bacteriostatic or bactericidal effects on Kp-2H7. Liquid chromatography-mass spectrometry (LC-MS) revealed that mice colonized with effective consortia (F31- or F18-mix) contained higher caecal levels of 4-hydroxybenzoic acid (4-HBA), cholic acid, acetate, and butyrate than did mice colonized with less-effective consortia (F13-mix, or F18-mix minus phylogenetic groups A, B, C, or D) or Kp-2H7 alone (**Extended Data Fig. 6b**). These molecules have previously been implicated in colonization resistance mechanisms against *Enterobacteriaceae*^26–28^. In our hands, however, 4-HBA, acetate, and butyrate exerted only weakly suppressive effects, requiring a high concentration of 100 mM (and, in the case of acetate and butyrate, low pH) to inhibit Kp-2H7 growth *in vitro*. Cholic acid was completely ineffective at all concentrations tested (**Extended Data Fig. 6c**). Furthermore, butyrate supplementation via tributyrin feeding did not significantly enhance the suppressive activity of F18-mix *in vivo* (**Extended Data Fig. 6d**).

We next examined whether F18-mix was acting by modulating processes associated with *Enterobacteriaceae* intestinal fitness. However, the deletion of Kp-2H7 genes associated with quorum sensing (*lsrA, lsrC, lsrB, lsrD, lsrR*)^29^, biofilm formation (*csgD*)^30,31^, stress response (*rpoS*)^30^, and nitrate respiration (*narG, narZ*)^1,8^ did not significantly affect sensitivity to F18-mix-mediated decolonization (**Extended Data Fig. 7a, b**). These results suggest that F18-mix exerts anti-*Klebsiella* activity through mechanisms that have not been well-characterized.

## F18-mix suppresses *Klebsiella* via gluconate competition.

**Figure 3:**
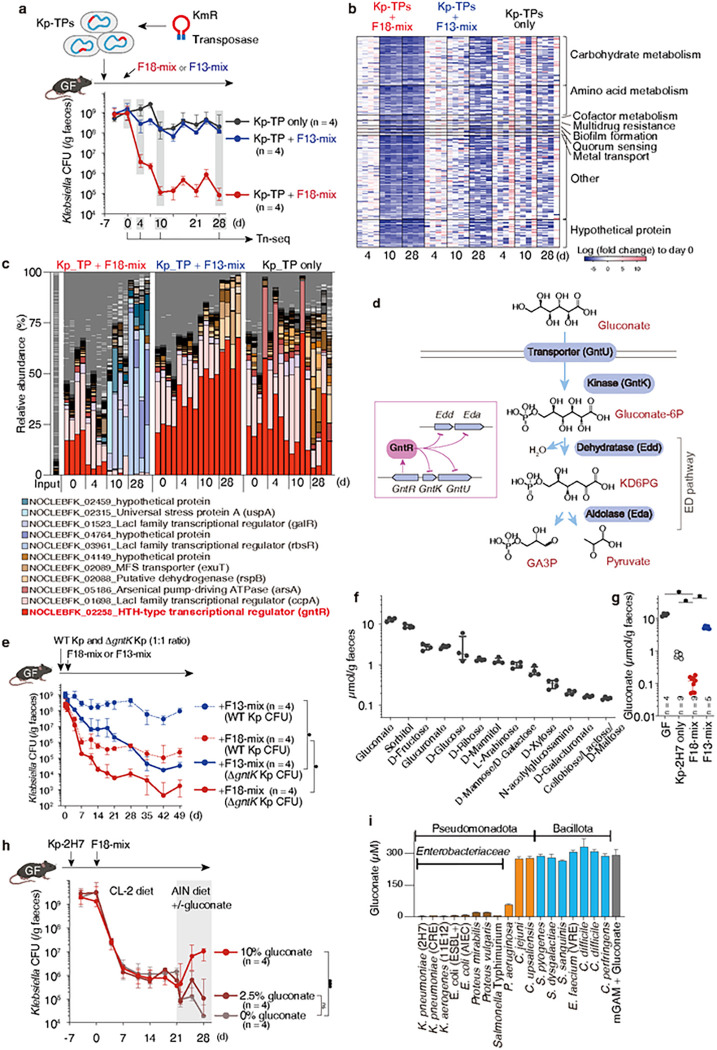
F18-mix competes with Kp-2H7 for gluconate. **a,** Using random mutagenesis with the Tn5-based transposon EZ-Tn5, 8 × 10^5^ kanamycin resistant (KmR) Kp-2H7 mutants (Kp-TPs) were obtained. Kp-TPs were pooled together and administered to GF mice, followed by oral administration of F18-mix or F13-mix. Faecal samples were collected and sequenced at day 0, 4, 10, and 28. **b**, Heatmap shows 194 Kp-2H7 genes that were significantly downregulated on day 10 post-F18-mix administration. **c**, Relative abundance of Kp-TP mutants in each mouse (4 mice per group). Mutants representing >15% of the total reads in any samples are noted in the legend. **d**, Gluconate metabolic pathway in *K. pneumoniae*. GntR suppresses expression of genes encoding gluconate transporter (*gntU*), gluconate kinase (*gntK*), and Entner-Doudoroff pathway enzymes (*edd* and *eda*). **e**, GF mice were colonized with a 1:1 mixture of wild-type (WT) and D *gntK* Kp-2H7, followed by oral administration of F18-mix or F13-mix. Faecal Kp-2H7 CFUs are shown. Data are expressed as the median ± IQR, representative from two independent experiments. *P < 0.05; by Mann-Whitney U test at last day. **f**, LC-MS/MS analysis of the indicated carbon source in faeces of GF mice fed a nutrient-rich diet (CL-2). **g**, Faecal gluconate levels in uncolonized GF mice or GF mice colonized with Kp-2H7, F18-mix, or F13-mix. Data are expressed as the median ± IQR. *P < 0.05; by Kruskal-Wallis test using the Benjamini-Hochberg for multiple comparisons. **h**, GF mice were monocolonized with Kp-2H7, followed by oral administration of F18-mix. Diet was switched from CL-2 to AIN93G supplemented with or without gluconate at day 21. Faecal Kp-2H7 CFUs are shown as median ± IQR, representative from two independent experiments. *P < 0.05; ns, not significant; by Kruskal-Wallis test using the Benjamini-Hochberg correction for multiple comparisons at day 28. **i**, Pathogenic strains were incubated with 300 mM gluconic acid in mGAM broth for 48 hours. Gluconate concentration in the culture supernatant was measured by LC-MS/MS in n = 3 biological replicates.

We next profiled the *Klebsiella* transcriptome in mice colonized with Kp-2H7 versus Kp-2H7+F18-mix. Co-colonization with F18-mix significantly altered the Kp-2H7 transcriptional landscape, particularly pathways involved in carbon metabolism, amino acid metabolism, and the phosphotransferase system (**Extended Data Fig. 8a, b**), suggesting competition for nutrients and substrates, consistent with previous studies showing that nutrients affect intestinal colonization of *Enterobacteriaceae*^12,32–35^. To identify Kp-2H7 genes that affect intestinal fitness upon co-colonization with F18-mix, we employed a transposon mutagenesis approach. We generated a highly saturated transposon mutant library with approximately 8 × 10^5^ distinct mutations in Kp-2H7, which is estimated to contain 100+ mutants per gene (Fig. 3a). GF mice were inoculated with a Kp-2H7 pool containing all transposon mutants (Kp-TPs) followed by oral gavage with F18-mix or F13-mix, and faecal samples were collected longitudinally and subjected to transposon insertion sequencing (Tn-seq)^36,37^ (Fig. 3a). After treatment with F18-mix, 194 Kp-2H7 mutants displayed significantly reduced fitness, many of which were deficient in carbohydrate and amino acid metabolism (Fig. 3b, **Table S2**). Specifically, mutations in genes involved in the metabolism of gluconate, glucose, fructose, and galacturonate accelerated Kp-2H7 decolonization in F18-mix-treated mice relative to untreated and F13-mix-treated mice (**Table S2**), suggesting that F18-mix competes with Kp-2H7 for these carbon sources.

Comparing the relative abundance of Kp-TPs within each mouse revealed that those deficient in the HTH-type transcriptional regulator gene (*gntR*) were cleared by day 10 in F18-mix-treated mice, but persisted at high levels in untreated and F13-mix-treated mice (Fig. 3c). GntR functions as a gluconate operon repressor^38,39^. In *Klebsiella* and other *Enterobacteriaceae* strains, gluconate is first phosphorylated by gluconate kinase (GntK/IdnK) to 6-phosphogluconate (gluconate-6P), which is in turn reduced to 2-keto-3-deoxy-6-phosphogluconate (KD6PG) by gluconate-6P dehydratase (Edd), and eventually converted into pyruvate and glyceraldehyde 3-phosphate (GA3P) by KD6PG aldolase (Eda) [Entner-Doudoroff (ED) pathway^40^, Fig. 3d]. GntR represses genes encoding gluconate transporter (*gntU*), gluconate kinase (*gntK/idnK*), and ED pathway enzymes (*edd* and *eda*) in *Klebsiella* when environmental gluconate is scarce^38,39^ (Fig. 3d). Therefore, the differential responses of *gntR* mutants *in vivo* suggest that gluconate metabolism mechanistically contributes to F18-mix-mediated anti-*Klebsiella* activity. Supporting this hypothesis, transcriptomic analysis of Kp-2H7 genes in faecal samples revealed severe suppression of gluconate operon genes (*gntK, gntU*, and *edd*) following treatment with F18-mix, but not F13-mix (**Extended Data Fig. 8c**). We generated *gntR*- and *gntK*-deficient isogenic Kp-2H7 mutants and examined their fitness *in vitro* and *in vivo* (**Extended Data Fig. 9a**). As expected, while the growth curves of both mutants *in vitro* were indistinguishable from wild-type (WT) Kp-2H7 in glucose-supplemented minimal media, D *gntR* Kp-2H7 growth was accelerated and D *gntK* Kp-2H7 growth was stunted relative to WT Kp-2H7 when cultured with gluconate as the sole carbon source (**Extended Data Fig. 9b**). We inoculated GF mice with a 1:1 mixture of either WT+D *gntR* or WT+D *gntK* Kp-2H7 and treated with F18-mix or F13-mix. The D *gntR* mutant was just as resistant to F13-mix treatment as WT Kp-2H7, but exhibited enhanced sensitivity to F18-mix treatment over WT Kp-2H7 (**Extended Data Fig. 9c**). Notably, D *gntK* Kp-2H7 showed a 3-log reduction following treatment with the otherwise less effective F13-mix (Fig. 3e). These results support the notion that controlling gluconate metabolism is one of the mechanisms involved in the suppression of *Klebsiella* by F18-mix.

To further investigate the importance of gluconate, we next assessed the concentration of various carbon sources in the faeces of GF mice by quantitative LC-MS and found that gluconate was the most abundant (Fig. 3f). Compared to GF mice, gluconate concentration dropped by 1 log in Kp-2H7-monocolonized mice and by 2 logs in F18-mix-colonized mice, but did not change substantially in F13-mix-colonized mice (Fig. 3g). Dietary gluconate supplementation significantly diminished the anti-*Klebsiella* suppressive effect of F18-mix (Fig. 3h), whereas gluconate deprivation reduced Kp-2H7 load in F18-mix-treated mice (Fig. 3h) and in monocolonized mice (**Extended Data Fig. 10a**). When Kp-2H7-monocolonized mice were sequentially inoculated with one of the F18 strains every five days over a 95-day period, we observed a cumulative decline in faecal gluconate that mirrored the reduction in Kp-2H7 burden (**Extended Data Fig. 10b, c**). Together, these results indicate that F18-mix suppresses *Klebsiella* through community action-mediated competitive reduction in gluconate availability, though we cannot exclude the contribution of additional factors.

Notably, gluconate preference is a relatively specific feature of Enterobacteriaceae (*Klebsiella* and *Escherichia*), as the tested *Pseudomonas, Campylobacter, Enterococcus faecium, Clostridioides difficile*, and *Clostridium perfringens* strains failed to efficiently consume gluconate *in vitro* (Fig. 3i), potentially explaining why F18-mix treatment was selectively effective against *Enterobacteriaceae* (Fig. 2a).

## Gluconate pathway genes in patients with IBD

**Figure 4:**
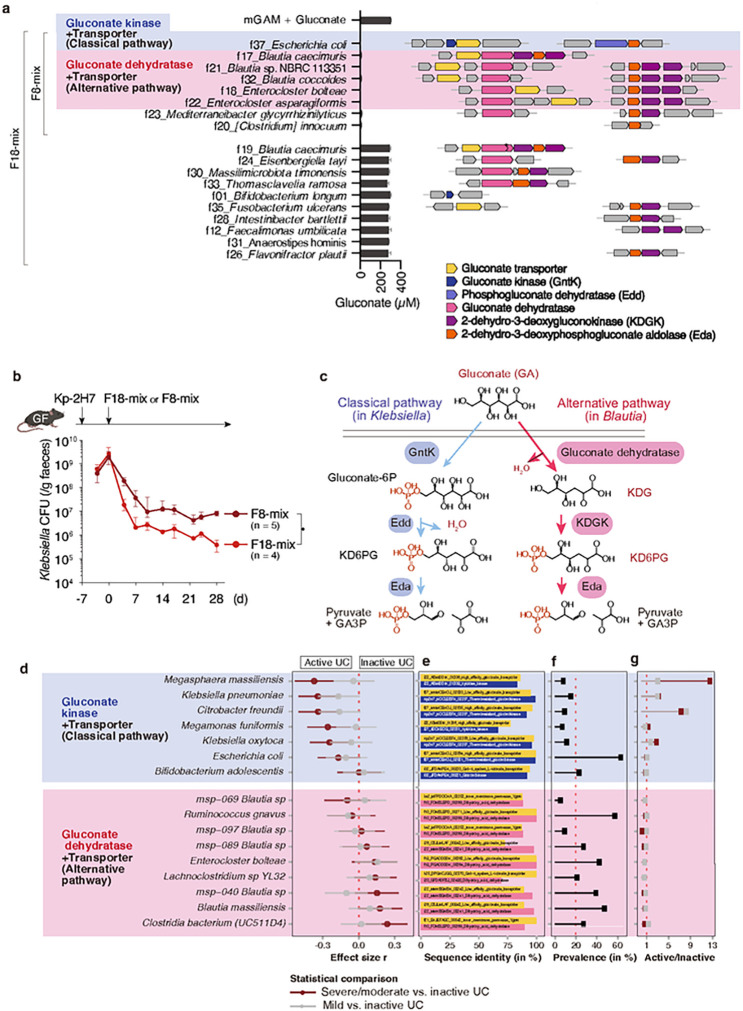
Association of gluconate pathway genes with IBD. **a**, *In vitro* gluconate consumption capabilities of the F18 strains are shown in the middle bar graph (n = 3 biological replicates). Genome neighbourhood of putative gluconate metabolism genes identified in the F18 strains are shown in the right. Asterisk indicates a non-functional frameshift mutation. **b**, GF mice were monocolonized with Kp-2H7 and treated with F8- or F18-mix. Faecal Kp-2H7 CFUs are shown. Data are expressed as the median ± IQR, representative from two independent experiments. *P < 0.05; by Mann-Whitney U test at day 28. **c**, Classical and alternative gluconate metabolism pathways typically carried by *Klebsiella* and *Blautia* species. **d**, Comparative analysis of the abundance of species containing gluconate-related genes among paediatric patients with ulcerative colitis (UC), classified as moderate/severe versus inactive disease (maroon) or mild versus inactive disease (grey). Species were grouped based on the combination of gluconate-related genes in their MSP bins. **e**, Overview of the sequence identity and sequence ID for gluconate-related genes per MSP. **f**, MSP prevalence across the cohort, shown as a percentage. **g**, Disease ratio of MSP prevalence between either moderate/severe vs. inactive disease (maroon) or mild vs. inactive disease (grey).

Of the F18 strains, 8 effectively consumed gluconate *in vitro* (Fig. 4a). A mixture of these 8 gluconate-utilizing strains (F8-mix) substantially reduced Kp-2H7 load in a gnotobiotic setting, though to a slightly lesser extent than did the full F18-mix (Fig. 4b), further supporting the gluconate competition hypothesis. We examined the genomes of F18 strains (**Table S1**) and found that 3 *Blautia*, 2 *Enterocloster*, and 1 *E. coli* strains carry gene clusters encoding enzymes and transporters putatively involved in gluconate metabolism (Fig. 4a, **Extended Data Fig. 11**). In contrast to the “classical” gluconate kinase-dependent metabolic pathway found in *Enterobacteriaceae*, the gene clusters identified in the *Blautia* and *Enterocloster* strains encode an “alternative” pathway that utilizes gluconate dehydratase. In this pathway, gluconate is first dehydrated to 2-keto-3-deoxygluconate (KDG), then phosphorylated into KD6PG by KDG kinase (KDGK), and eventually cleaved into pyruvate and GA3P by Eda^41,42^ (Fig. 4c). We next queried the presence of alternative gluconate pathway genes in the genomes of our donor F-, K-, and I-derived culture collections (comprising 101 isolates) (**Table S1**). Classical gluconate operon genes were identified in several *Enterobacteriaceae, Bifidobacterium*, and *Megasphaera* species, whereas alternative gene clusters encoding both gluconate transporter and gluconate dehydratase homologues were identified in *Blautia, Ruminococcus, Enterocloster*, and *Faecalibacterium* species (**Extended Data Fig. 11, Table S3**). The carriage of gluconate pathway gene clusters, but not the transporter or dehydratase/kinase alone, was associated with effective gluconate consumption *in vitro* (**Extended Data Fig. 11**).

Finally, we examined faecal gluconate levels as well as the abundance and prevalence of species carrying gluconate operon genes in paediatric patients with UC in active (mild versus moderate to severe) and inactive states from the PROTECT cohort^43^ (**Extended Data Fig. 12a**). Intensity of faecal gluconate (C_6_H_12_O_7_) as predicted by LC-MS was used as a proxy for intestinal concentration, and was positively associated with levels of faecal calprotectin (**Extended Data Fig. 12b**). Supporting the idea that gluconate confers improved fitness to *Enterobacteriaceae*, metagenomic species (MSPs) within *Citrobacter freundii, Klebsiella oxytoca, K. pneumoniae, and E. coli* that carry gluconate kinase and transporter genes were significantly more prevalent and abundant in UC patients in active versus inactive disease states (Fig. 4d–g). In addition, classical gluconate operon-encoding *Veillonellaceae* and *Selenomonadaceae* family members, such as *Megasphaera massiliensis* and *Megamonas funiformis*, were significantly enriched in UC patients with moderate/severe versus inactive disease (Fig. 4d–g). In contrast, alternate gluconate operon-encoding *Blautia, Clostiridium*, and *Faecalibacterium* were more abundant in patients in inactive disease states. We also examined the adult IBD cohort HMP2 (ref. ^14^) (**Extended Data Fig. 12a**). IBD was associated with expansion of gluconate kinase gene-carrying *Enterobacteriaceae* species (**Extended Data Fig. 12c**). In particular, *E. coli, C. freundii*, and *K. pneumoniae* consistently emerged as significantly more prevalent in disease in the HMP2 cohort. Overall, enrichment of gluconate operon genes in proinflammatory *Enterobacteriaceae* is associated with active disease and may contribute to IBD flares, whereas enrichment of alternative pathways in commensals is associated with disease remission and may enable nutrient competition that ultimately suppresses proinflammatory pathobionts and ameliorates gastrointestinal pathology.

## Discussion

*Enterobacteriaceae* species, such as *K. pneumoniae* and *E. coli*, have been linked to both IBD-associated inflammatory pathology as well as multidrug-resistant nosocomial infections for which there are limited therapeutic options^3,4^. Broad-spectrum antibiotics are often employed to treat multidrug-resistant *Enterobacteriaceae*, which may further aggravate dysbiosis and thus impair colonization resistance. In this study, we adapted a top-down gnotobiotic approach^44^ to elaborate a defined microbial consortium consisting of 18 effector bacterial strains from a healthy individual, which is capable of decolonizing *Enterobacteriaceae* strains. This F18-mix exerts potent anti-*Enterobacteriaceae* effects, likely through multiple mechanisms including restriction of nutrient availability and reshaping of ecological niches within the intestine. Each microbiota member possesses a unique nutritional program, which in turn determines local nutrient availability and thus niche definition. Our results, together with previous studies^35,45^, suggest that *Enterobacteriaceae* has a hierarchy of carbon preferences with gluconate being one of the most-preferred carbon sources in the intestine. When faced with F18-mix-mediated gluconate restriction, *Klebsiella* compensates by metabolically switching to utilize other unpreferred carbon sources. However, it is likely that F18-mix can effectively consume several of these alternative carbon sources as well, thereby further restricting nutrient availability to *Klebsiella*. Although more research will be needed to fully elucidate the rules governing effective competition (including other nutritional dependencies and the role of interspecies cooperation, among others), our findings establish a solid foundation for the development of microbiota-directed therapeutics to suppress *Enterobacteriaceae* pathobionts via ecological control. Overall, the F18-mix strains represent a promising candidate for clinical development as a rationally-defined microbial consortia to treat prevalent infectious and inflammatory diseases.

## Materials And Methods

### Mice

C57BL/6 mice, maintained under specific-pathogen-free or germ-free (GF) conditions, were purchased from Sankyo Laboratories Japan, SLC Japan, or CLEA Japan. GF and gnotobiotic mice were bred and maintained within the gnotobiotic facility of Keio University School of Medicine or the JSR-Keio University Medical and Chemical Innovation Center. *Il10*^−/−^ and *Ifngr1*^−/−^ mice were purchased from Jackson Laboratories. *Myd88*^−/−^*Trif*^−/−^ and *Rag2*^−/−^*gc*^−/−^ mice were purchased from Oriental Bio Service, Japan. All animal experiments were approved by the Keio University Institutional Animal Care and Use Committee.

### Human faecal samples and isolation of bacterial strains

Human faecal samples were obtained from healthy human donors, patients with ulcerative colitis (UC), and patients with Crohn’s disease (CD) following the protocol approved by the Institutional Review Board of Keio University School of Medicine (approval numbers #20150075, #20140211, and #20150075). Informed consent was obtained from each individual. Faecal samples were mixed with PBS (containing 20% glycerol) and stored at −80°C. An aliquot of each sample was diluted with PBS in an anaerobic chamber (80% N_2_, 10% H_2_, and 10% CO_2_; Coy Laboratory Products) and plated onto different agar plates (EG, mGAM, BHK, CM0151, MRS, or BL). After incubating for 2–7 days, colonies with different appearances were transferred to liquid media (EG, mGAM, HK, or CM0149), incubated for 24–48 hours, mixed with glycerol [final concentration 20% (v/v)], and stored at −80°C. Bacterial genomic DNA was extracted from the isolated strains using the same protocol as DNA isolation from faecal samples (below). The 16S rRNA was amplified by PCR using the KOD plus Neo kit (TOYOBO) according to the manufacturer’s protocol. DNA sequencing was performed by Eurofins. Sequences were aligned using the BLAST program of NCBI and the Ribosomal Database Project (RDP) databases. Primers used for DNA sequencing were as follows: F27 primer: 5’-AGRGTTTGATYMTGGCTCAG-3’; R1492 primer: 5’-TACGGYTACCTTGTTACGACTT-3’. Individual isolates in the culture collection were grouped as “strains” if their 16S rRNA gene sequences shared >98.0% homology.

To prepare the bacterial mixture for inoculation, isolated strains were individually cultured in the appropriate broth at 37°C for 1–2 days (mGAM broth was used for culturing the F18 strains). Bacterial density was adjusted based on absorbance at 600 nm values, and equal volumes of the cultured strains were mixed and centrifuged at 3000 × *g* for 10 min at 4°C to concentrate fivefold. Thereafter, GF mice were administered 200 mL of the bacterial mixture/mouse (approximately 1–2 × 10^9^ CFU of total bacteria) by oral gavage. The bacterial mixture was administered into GF mice (200 mL/mouse, approximately 1–2 × 10^9^ CFU of total bacteria) by oral gavage.

### Colonization of mice with pathogenic bacterial strains

C57BL/6 GF mice (8–14 weeks of age, housed in separate GF isolators) were inoculated with *Klebsiella pneumoniae* 2H7 (Kp-2H7), carbapenem-resistant *Klebsiella pneumoniae* (CPM^+^ Kp, ATCC BAA1705), *Klebsiella aerogenes* (strain Ka-11E12, ref. ^17^), extended-spectrum-b-lactamase producing *E. coli* (ESBL^+^ *E. coli*, ATCC BAA2777), adherent-invasive *E. coli* (AIEC, strain LF82, provided by Nicolas Barnich^22^), *Pseudomonas aeruginosa* (ATCC 10145), Vancomycin-resistant *Enterococcus faecium* (VRE Ef, ATCC 700221), *Campylobacter upsaliensis* (ATCC BAA1059), or *Clostridioides difficile* (strain 630, ATCC BAA1382) by oral gavage (2 × 10^8^ CFU//mouse). Seven days after colonization with pathogenic microbes, the mice were administered 200 mL of human faecal suspension or 200 mL of isolated bacterial strain mix (total 10^9^ CFU) by oral gavage. Faecal samples were collected from mice every two or three days, suspended in PBS (containing 20% glycerol), and cultured on selective media [DHL agar with 30 mg/L ampicillin and 30 mg/L spectinomycin for Kp-2H7, CRE Kp, Ka-11E12, and *P. aeruginosa*, MacConkey agar with 1 mg/L cefotaxime, and VRE-selective agar plates (BD #251832) for VRE]. After 24–48 hours of incubation, the CFUs were counted. In cases where evaluation was not possible by counting CFUs, bacterial DNA extracted from faeces was evaluated by quantitative real-time PCR (qPCR). Unless otherwise stated, mice were fed a high-calorie diet (CL-2; CLEA Japan, Inc.). To evaluate the effect of dietary gluconate supplementation, a chemically defined diet (AIN93G; Oriental Yeast Co., Ltd) supplemented with 0%, 2.5% or 10% gluconate was used. To examine the effectiveness of each of the 18 strains, C57BL/6 GF mice were inoculated with Kp-2H7 (2 × 10^8^ CFU/mouse) by oral gavage, followed by oral administration of each strain of the F18-mix one by one every five days for 95 days. Faecal samples were collected every five days to count the CFU of Kp-2H7 as well as to quantify the levels of gluconate.

### Bacterial DNA extraction, quantitative real-time PCR, and 16S rRNA gene sequencing

The frozen faecal samples were thawed and 50 μL of each sample was mixed with 350 μL TE10 (10 mM Tris-HCl, 10 mM EDTA) buffer containing RNase A (final concentration 100 μg/mL, Invitrogen) and lysozyme (final concentration 3.0 mg/mL, Sigma). The suspension was incubated for one hour at 37°C with gentle mixing. Purified achromopeptidase (Wako) was added to a final concentration of 2,000 unit/mL, and the sample was further incubated for 30 min at 37°C. Then, sodium dodecyl sulphate (final concentration 1%) and proteinase K (final concentration 1 mg/mL, Nacalai) were added to the suspension and the mixture was incubated for one hour at 55°C. Thereafter, purified DNA was obtained from the samples using the Maxwell^®^ RSC cultured cell DNA kit, according to the manufacturer’s protocol. For quantifying the amount of bacterial DNA, real-time qPCR was performed using the Thunderbird SYBR qPCR Mix (TOYOBO) and LightCycler 480 (Roche). The primer pairs used in this study are listed in **Table S4**.

16S rRNA gene sequencing was performed using MiSeq according to the Illumina protocol. PCR was performed using primers 27Fmod (5’-AGRGTTTGATYMTGGCTCAG-3’) and 338R (5’-TGCTGCCTCCCGTAGGAGT-3’) to amplify the V1–V2 region of the 16S rRNA gene. Amplicons (approximately 330 bp in size) generated from each sample were purified using AMPure XP magnetic beads (Beckman Coulter). DNA was quantified using the Quant-iT Picogreen dsDNA assay kit (Invitrogen) and Infinite M Plex plate reader (Tecan), according to the manufacturer’s instructions, and then stored at 4°C. The pooled amplicon library was sequenced using the MiSeq Reagent Kit v2 (500 cycles) and MiSeq sequencer (Illumina; 2 × 250-bp paired-end reads). After demultiplexing the 16S sequence reads based on the sample-specific index, primer sequences were trimmed by Cutadapt v. 3.3^46^. The trimmed reads were uploaded to the DADA2 R package v.4.0.3 (ref. ^47^) to construct amplicon sequence variants (ASVs) using the filterAndTrim function with the following parameters: maxN = 0, truncQ = 2, maxEE = 2, and truncLen = c (200,180). Possible chimeric reads were removed with the removeBimeraDenovo function of the DADA2. The taxonomic assignment of each ASV was determined by similarity searching using the GLSEARCH program. For determining taxonomy of ASV sequences that originated from human faecal samples, 16S RefSeq from NCBI, RDP^48^, CORE^49^, and GRD (https://metasystems.riken.jp/grd/) were used as the reference database. The sequences of isolates were compared to ASV detected in the faecal microbiome of donors F, I, and K, and those matching >99% were determined to be their corresponding ASVs.

### Bacterial whole-genome sequencing

The Illumina MiSeq and PacBio Sequel platforms were used for bacterial whole-genome sequencing. For Illumina sequencing, the library was prepared using the TruSeq DNA PCR-free library prep kit (Illumina), with a target insert size of 550 bp. All the Illumina reads were trimmed and filtered using the FASTX-toolkit (version 0.0.13). For the PacBio sequencing, the library was prepared using the SMRTbell template prep kit 1.0. Sequence data for both types of sequencing were assembled using the hybrid assembler Unicycler. Taxonomic assignment of the genomes was determined by classify_wf of GTDB-tk^50^ version 2.3.0 with GTDB^51^ database R214. NCBI taxonomy of fastANI reference genome related to the genome of each strain was retrieved using NCBI-genome-download version 0.3.3 (ncbi-genome-download; DOI: 10.5281/zenodo.8192432) and rankedlineage.dmp from NCBI taxonomy database^52^ (downloaded on 14/09/2023). The genes were predicted using Prokka version 1.14.0 with “--kingdom Bacteria --rnammer” options, and rnammer version 1.2. The homology search for the predicted genes was performed using diamond^53^ version 2.0.15 with “blastp --evalue 0.00001 --id 30 --query-cover 60 --ultra-sensitive” options, with KEGG (downloaded on 19/04/2022)^54^, COG (downloaded on 19/05/2021)^55^, VFDB (downloaded on 10/09/2022)^56^, and UniRef90 (downloaded on 24/05/2022; https://www.uniprot.org/help/uniref) databases. For homology search against KEGG DB, a database was manually constructed from protein sequences with KEGG Ontology (K number) which were extracted from KEGG non-redundant datasets at the species level. We also added homology search for gluconate metabolism genes in our isolated strains with “blastp --evalue 0.00001 --id 20 --query-cover 60 --ultra-sensitive” options. The sequences of gluconate kinase (*gntK*, MKMCEHOJ_02531) and gluconate transporters (MKMCEHOJ_02530 and MKMCEHOJ_02505) from f37_*E. coli* strain, and gluconate dehydratase (EAOGLLOI_00767), gluconate transporters (EAOGLLOI_00766 and EAOGLLOI_00912), 2-dehydro-3-deoxygluconokinase (*kdgK*, EAOGLLOI_00768), and 2-dehydro-3-deoxyphosphogluconate aldolase (*eda*, EAOGLLOI_00769) from f17_*Blautia caecimuris* strain were used as reference sequences.

### *Ex vivo* caecal suspension culture

Caecal contents from GF or F31-, F18-, and F13-mix colonized mice were anaerobically resuspended in water at a concentration of 100 mg/mL. Caecal contents were either filtered through a 0.22 μm filter (Millex Millipore) after centrifuging at 10,000 × *g* for 5 min, heat-killed at 105 °C for 30 min, or left untreated. Thereafter, a diluted overnight culture of Kp-2H7 (10^3^ CFU in 10 μL) was added to 200 μL of each caecal suspension. After incubating at 37°C for 48 hours under aerobic or anaerobic conditions, samples were serially diluted and plated on a selection agar plate (DHL with 30 mg/L ampicillin and 30 mg/L spectinomycin) for counting Kp-2H7 CFU.

### Bacterial growth monitoring

The wild type, Δ*gntK*, or Δ*gntR* Kp-2H7 strain was cultured in M9 minimal medium for 24 hours at 37°C, which was diluted 100 times with sterile water. A 10 μL culture dilution was inoculated into 200 μL of M9 medium with 0.4% of glucose or gluconate as the sole carbon source or without carbon. To examine the effect of metabolites on Kp-2H7 growth, 10 mL of Kp-2H7 culture dilutions were inoculated into 200 μL of M9 medium containing varying concentrations of 4-HBA (4-hydroxybenzoic acid) (100, 10, 1, or 0.1 mM), cholic acid (500, 100, 20, or 4 μM), and acetate or butyrate (100, 25, 6.25, 1.56 or 0.39 mM). The pH of acetate and butyrate was adjusted to either 5.0 or 7.0. Bacterial growth was monitored by measuring absorbance at 600 nm every 30 minutes using a microplate reader [Sunrise Thermo (Tecan) for anaerobic conditions and Infinite 200 PRO (Tecan) for aerobic conditions] at 37°C with a 100-second shaking before each time point.

### Transcriptome analysis of epithelial cells

Total RNA was isolated from colonic epithelial cells using NucleoSpin RNA (MACHEREY-NAGEL), according to the manufacturer’s instructions. Libraries for RNA sequencing were prepared using TruSeq Stranded mRNA Library Prep (Illumina Inc.), according to the manufacturer’s instructions. The libraries were sequenced using NovaSeq 6000 (Illumina Inc.) with the mode of 150-bp paired-end. The sequenced paired-end reads were quality-controlled using Trimmomatic^57^ version 0.39 with “2:30:10 LEADING:3 TRAILING:20 SLIDINGWINDOW:4:15 MINLEN:5” options and FASTX-Toolkit version 0.0.13 (http://hannonlab.cshl.edu/fastx_toolkit/index.html) with “-q 20 -p 80” options. Unpaired reads were excluded from further analyses. The remaining quality-controlled reads were mapped to the mouse reference genome (mm10) using STAR^58^ version 2.7.2b. The mapped reads were counted for each gene using featureCounts^59^ version 1.5.2 with “-t exon -p -B -Q 1” options. the transcripts per million (TPM) values of each gene in each sample were calculated. The differential expression analysis was performed using DESeq2^60^ version 1.28.1, and the p-values were corrected by Benjamini-Hochberg (BH) method to maintain the false discovery rate (FDR) below 5%.

### Transcriptome analysis of Kp-2H7

Total RNA was isolated from faecal samples using NucleoSpin RNA (MACHEREY-NAGEL), according to the manufacturer’s instructions. Libraries for RNA sequencing were prepared using TruSeq Stranded mRNA Library Prep (Illumina Inc.) and sequenced using HiSeq X (Illumina Inc.) with the mode of 150-bp paired-end. To analyse the transcriptome profiles of Kp-2H7 in the presence or absence of F18-mix, a reference genome was created by concatenating the genome sequence of Kp-2H7 with the genome sequences of the F18-mix. The sequenced paired-end reads were quality-controlled using Trimmomatic^57^ version 0.39 with “2:30:10 LEADING:3 TRAILING:20 SLIDINGWINDOW:4:15 MINLEN:5” options and FASTX-Toolkit version 0.0.13 (http://hannonlab.cshl.edu/fastx_toolkit/index.html) with “-q 20 -p 80” options. Unpaired reads were excluded from further analyses. The remaining reads were mapped to the mouse (mm10) and PhiX reference genomes using minimap2 version 2.17-r941 with “-N 1 -a” options^61^. Then, the reads unmapped to the mouse genome were extracted to obtain quality-controlled reads for subsequent analyses. The quality-controlled reads were mapped to the concatenated reference genome using bowtie2^62^ version 2.3.4.1. Uniquely mapped reads were counted for each Kp-2H7 gene. The differential expression analysis was performed using DESeq2^60^ version 1.28.1 with BH-correction method to maintain the FDR below 5%. The heatmap was obtained from the variance-stabilizing transformations values obtained from the DESeq2 output.

For real-time qPCR analysis, cDNA was synthesized using ReverTra Ace qPCR RT Master Mix (TOYOBO), and qPCR was performed using Thunderbird SYBR qPCR Mix (TOYOBO) on a LightCycler 480 (Roche).

### Construction of transposon mutant library

A transposon insertion library of Kp-2H7 was constructed using the EZ-Tn5TM <KAN-2> Tnp TransposomeTM kit (Lucigen Corp, USA). Briefly, 80 μL (10^9^ CFU) of Kp-2H7 suspension was mixed with 0.5 μL of EZ-Tn5TM <KAN-2>, transferred to a 1-mm gap width electroporation cuvette, and subjected to electroporation using ELEPO21 (Nepa Gene Co. Ltd., Japan) with the following parameters: poring pulse; voltage: 1800 V, pulse length: 5.0 msec, pulse interval: 50 msec, number of pulses: 1, and polarity: +, and transfer pulse; voltage: 150 V, pulse length: 50 msec, pulse interval: 50 msec, number of pulses: 5, and polarity: ±. Transformed Kp2H7 cells were incubated in 1 mL of LB broth for three hours at 37°C, and then selected on LB agar plates containing kanamycin (90 mg/L) at 37°C. Thereafter, approximately 8 × 10^5^ transposon mutant colonies were collected and stored at −80°C in LB containing 20% glycerol.

### Transposon sequencing

GF mice were colonized with the pool of 8 × 10^5^ Kp-2H7 transposon mutants. Faecal samples were collected on day 0, 4, 10, and 28 following colonization, suspended in PBS (50 mg/mL) containing 20% glycerol, and cultured overnight at 37°C on LB agar plates containing kanamycin (90 mg/L). Kp-2H7 mutant colonies were scraped together and DNA was extracted by the method described above. Transposon sequencing was carried out according to the method described by Kazi et al.^63^. Briefly, genomic DNA was fragmented via sonication. Then, a poly-C tail was added to the 3’ end of the DNA fragment by terminal deoxynucleotidyl transferase. The transposon junctions were amplified using a biotinylated primer, which was then enriched using streptavidin beads. By performing a second nested PCR, a single barcode was added to each sample. The libraries were sequenced using HiSeq 2500 (Illumina Inc.) with the mode of 50-bp single-end. The sequenced reads were quality-controlled using Trimmomatic^57^ version 0.39 with “2:30:10 LEADING:3 TRAILING:20 SLIDINGWINDOW:4:15 MINLEN:5” options and FASTX-Toolkit version 0.0.13 (http://hannonlab.cshl.edu/fastx_toolkit/index.html) with “-q 20 -p 80” options. Unpaired reads were excluded from further analyses. The remaining reads were mapped to the mouse reference genome (mm10) using minimap2 version 2.17-r941 with “-N 1 -a -x sr” options^61^. Then, the reads unmapped to the mouse genome were extracted to obtain quality-controlled reads for subsequent analyses. The quality-controlled reads were mapped to the Kp-2H7 assembled genome using bowtie2 version 2.4.2. The mapped reads were counted for each gene using featureCounts3 version 1.5.2 with “-t CDS -p -B -Q 1” options, and the TPM of each gene was calculated as the relative abundance of a gene mutant in a sample by assuming that each transposon mutant has a single insertion. The differential abundance mutants were detected by Welch’s t-test for log-scaled TPM with BH-correction method to maintain the FDR below 5%.

### Generation of Kp-2H7 mutants

The Kp-2H7 deletion mutants of were generated using the Quick and Easy *E. coli* Gene Deletion Kit (Gene Bridges, Heidelberg) according to the manufacturer’s protocol. Briefly, Kp-2H7 cells were transformed with the pRED/ET plasmid harbouring the tetracycline-resistant gene by electroporation. Bacteria with pRED/ET were selected on LB plates containing tetracycline (30 mg/L) at 30°C. Thereafter, these cells were incubated in LB broth with appropriate antibiotics at 30°C until absorbance at 600 nm reached 0.2, followed by an additional hour of incubation with 0.3% L-arabinose at 37°C to induce the expression of the recombinant proteins. These cells were used to prepare electrocompetent cells and were transformed with the linear DNA fragment (the FRT-PGK-gb2-neo-FRT cassette)-flanked homology arms. The functional cassettes were generated by PCR, according to the manufacturer’s protocol. The primers with homology arms are listed in **Table S4**. The electroporated cells were incubated in 1 mL of LB broth for three hours at 37°C. Gene deletion strains were selected on LB agar plates with kanamycin (90 mg/L) after overnight growth at 37°C. The double or triple knockout strains were generated by removing the kanamycin selection marker through electroporation of the FLP expression plasmid (707-FLPe) and repeating the above-mentioned protocol. The deletions were confirmed by DNA sequencing.

### Isolation of lymphocytes and flow cytometry

Lymphocytes were collected from the large intestines and analysed according to previously described protocols^17,64^. Briefly, the intestines were dissected longitudinally and washed with PBS to remove all luminal contents. All samples were incubated in 15 mL of Hanks’ balanced salt solution (HBSS) containing 5 mM EDTA for 20 min at 37°C in a shaking water bath to remove epithelial cells. Thereafter, after removal of any remaining epithelial cells, muscular layers and fat tissues using forceps, the samples were cut into small pieces and incubated in 10 mL of RPMI1640 containing 4% foetal bovine serum (FBS), 0.5 mg/mL collagenase D (Roche Diagnostics), 0.5 mg/mL dispase II (Roche Diagnostics), and 40 μg/mL DNase I (Roche Diagnostics) for 50 min at 37°C in a shaking water bath. Thereafter, the resultant digested tissues were washed with 10 mL of HBSS containing 5 mM EDTA, resuspended in 5 mL of 40% Percoll (GE Healthcare), and underlaid with 2.5 mL of 80% Percoll in a 15-mL Falcon tube. Percoll gradient separation was performed by centrifugation at 850 × *g* for 25 min at 25°C. Lymphocytes were collected from the interface of the Percoll gradient and washed with RPMI1640 containing 10% FBS, and then stimulated with 50 ng/mL PMA and 750 ng/mL ionomycin (both from Sigma) in the presence of Golgistop (BD Biosciences) at 37°C for four hours. After labelling of the dead cells with Ghost Dye Red 780 Viability Dye (Cell Signaling Technology), the cells were permeabilized and stained with anti-CD3e (BUV395; Biolegend), CD4 (BUV737; Biolegend), CD8a (PE/Cy7; Biolegend), TCRβ (BV421; Biolegend), and IFN-γ (FITC; Biolegend) using the Foxp3/Transcription Factor Staining Buffer Kit (Tonbo Biosciences), according to the manufacturer’s instructions. All data were collected on a BD LSRFortessa (BD Biosciences) and analysed using Flowjo software (TreeStar). CD4+ T cells were defined as a CD4+ TCRβ+ CD3e+ subset within the live lymphocyte gate.

### Measurement of lipocalin-2 and calprotectin

The faecal pellets from *Il10*^−/−^ mice were vortexed, suspended in PBS (5% w/v) with Complete Protease Inhibitor Cocktail (1 tablet dissolved in 50 mL PBS; Roche) and centrifuged, and supernatants were collected. The concentration of lipocalin-2 and calprotectin in faecal supernatants was measured by ELISA (Mouse Lipocalin-2 Matched Antibody Pair Kit; Abcam, Mouse S100A8/S100A9 Heterodimer DuoSet; R&D), according to the manufacturer’s protocol.

### Histological analysis

Colon tissue samples were dissected longitudinally and swiss-rolled, fixed with 4% paraformaldehyde, embedded in paraffin, sliced to 5μm sections and stained with hematoxylin and eosin. The degrees of colitis were graded by The Mouse Colitis Histology Index^65^. The histological slides were evaluated blind by two investigators.

### Non-targeted metabolomics analysis

C57BL/6 GF mice were monocolonized with Kp-2H7, followed by oral administration of bacterial mix. Caecal contents were collected on day 28 after administration of isolated bacterial mix and stored at −80°C until use. Frozen caecum contents were homogenized by shaking with metal corn using a multi beads shocker as previously described^66^. Then, the samples were suspended in 400 μL of methanol per 100 mg caecum content, and a 40 mL aliquot was subjected to the single layer extraction and untargeted LC-QTOF/MS analysis^66^. SCFAs were simultaneously extracted and derivatized from 20 μL of the suspension by using pentafluorobenzyl bromide alkylation reagent (Thermo Fischer Scientific, Waltham, MA, USA), and analysed by GCMS as previously described^67^. Water-soluble metabolites were extracted by first mixing 4 μL of the suspension, 196 μL of methanol, 200 μL of chloroform, 70 μL of water, and 10 μL of internal standards mix [100 μM of cycloleucine, 500 μM of citric acid-d4, and 1.0 mM of ornithine-d7 (Cambridge isotope laboratories, Andover, MA, USA)]. After vortexing for 1 min and centrifugation at 15000 × *g* for 5 min at 4°C, 100 μL of supernatant was evaporated to dryness. The dried samples were derivatized via methoxyamination, trimethylsilylation, or tert-butyldimethylsilylation, and then analysed by GC-MS/MS using Smart Metabolite DatabaseTM (Shimadzu Corp., Kyoto, Japan) or GC-MS operated in selected ion monitoring mode, as described previously^68^. Bile acids were extracted from 4 μL of the suspension mixed with deuterium-labelled internal standard mix [1.0 μM of cholic acid-d4, 1.0 μM of lithocholic acid-d4, 1.0 μM of deoxycholic acid-d4, 1.0 μM of taurocholic acid-d4, and 1.0 μM of glycocholic acid-d4 (Cayman Chemical)] using the Monospin C18 column (GL science). The column was washed with 300 μL of water (x2) and 300 μL of hexane (x1). Bile acids were eluted with 100 μL of methanol, then subjected to LC-MS/MS analysis using an UPLC I class (Waters) with a linear ion-trap quadrupole mass spectrometer (QTRAP 6500; AB SCIEX) equipped with an Acquity UPLC BEH C18 column (50 mm, 2.1 mm, and 1.7 μm; Waters). Samples were analysed with a mobile phase consisting of water/methanol/acetonitrile [14:3:3 (vol/vol/vol)] and acetonitrile, both containing 5 mM ammonium acetate, for 4 min, which was changed to 40:60 after 12 min, to 5:95 after 2 min, and then held for 2 min; with flow rates of 300 μL/min. Bile acids were detected by multiple-reaction monitoring in negative mode. Ions of [M-H]^−^, taurine (*m/z* = 124), and glycine (*m/z* = 74), generated from the precursor ion, were monitored as product ions for non-conjugated, taurine-conjugated, and glycine-conjugated bile acids, respectively. MS/MS settings were as follows: ion source, turbo spray; curtain gas, 30 psi; collision gas, 9 psi; ionspray voltage, −4500 V; source temperature, 600°C; ion source gas 1, 50 psi; and ion source gas 2, 60 psi.

### Measurement of carbohydrate levels

To evaluate bacterial gluconate utilization *in vitro*, isolated strains were cultured in mGAM broth or RCM containing 300 μM gluconic acid for 48 hours at 37°C under anaerobic conditions. Supernatant of each culture broth was collected, and the concentration of gluconate was measured by the ExionLC AD and SCIEX Triple Quad 6500+ LC-MS/MS system. To evaluate carbon level in faeces, each faecal sample was suspended in water (50 mg/mL), and the carbon levels in the culture supernatant were measured by LC-MS/MS. The measurement conditions for gluconate, glucuronate, and galacturonate were as follows: chromatographic separation was performed using the Intrada Organic Acid column, 150 × 2 mm (Imtakt); column temperature was 40°C; and the volume of each injection was 2 μL. The mobile phase comprising A (acetonitrile/water/formic acid = 10/90/0.1) and B (acetonitrile/100mM ammonium formate = 10/90) was used under gradient conditions: 0–3 min, A 100%, B 0%; 3–10 min, A 100%, B 0%; 10–13 min, A 0%, B 100%; 13–13.1 min, A 0%, B 100%; and 13.1–18 min, A 100%, B 0%); and the flow rate was 0.2 mL/min. Detailed MS conditions were as follows: curtain gas, 30 psi; Collision Gas, 9; ionSpray voltage, −4500 V; temperature, 400°C; ion source gas 1, 50 psi; and ion source gas 2, 80 psi. The retention time and Multiple reaction monitoring (MRM) transitions are listed in **Table S5**. The measurement conditions for other carbons were as follows: chromatographic separation was performed using the UK-Amino column (UKA26), 250 × 2 mm, (Imtakt); column temperature was 60°C and the volume of each injection was 2 μL. The mobile phase comprising A (10 mM ammonium acetate) and B (acetonitrile) was used under gradient conditions: 0–10 min, A 0%, B 100%; 10–50 min, A 0%, B 100%; 50–65 min, A 12%, B 88%; 65–70 min, A 60%, B 40%; 70–70.1 min, A 60%, B 40%; and 70.1–75 min, A 100%, B 0%); and the flow rate was 0.2 mL/min. Detailed MS conditions were as follows: curtain gas, 25 psi; collision gas, 9; ionspray voltage, −4500 V in negative mode and 5500 V in positive mode; temperature, 250°C, ion source gas 1, 50 psi; and ion source gas 2, 70 psi. Multiple reaction monitoring parameters are listed in **Table S5**. Data were obtained using Analyst software version 1.7.1 and analysed using SCIEX OS-MQ software version 2.1.0.55343.

### Metagenomic analysis of IBD cohorts

To systematically explore both established and novel microbial taxa possessing gluconate operon genes, gene catalogues were acquired from two distinct cohorts with IBD etiology: the paediatric PROTECT and adult HMP2 cohorts, comprising 240 and 1638 longitudinal metagenomic samples from 94 and 91 individuals, respectively. Metagenomic Species Pangenomes (MSPs) were constructed via the co-abundant gene binning approach (MSPminer^69^), followed by quality assessment (CheckM^70^), as described by Schirmer et al. (PROTECT)^3^ and Kenny et al. (HMP2)^71^. A targeted screening of these bins with DIAMOND BLASTP version 0.9.14^72^ was conducted to identify genes associated with gluconate transport and metabolism, retaining hits with an e-value <0.01 and sequence identity ≥60%. MSPs were categorized based on the combinations of gluconate-related genes detected. A differential abundance analysis was performed on TPM-normalized and Centred Log-Ratio-transformed MSP counts to control for sequencing depth, gene length, and compositional biases. Statistical significance was ascertained through a non-parametric Mann-Whitney U test accompanied by Benjamini-Hochberg correction. Effect sizes (r), calculated as the test statistic divided by the square root of the sample size, along with bootstrapped confidence intervals, were computed to account for unbalanced group sizes, offering insights into the robustness and directionality of the observed effects.

For the PROTECT cohort, comparative analyses were executed on randomly chosen cross-sectional samples from children manifesting mild UC (n = 32) or moderate to severe UC (n = 23), against inactive UC (n = 39). To validate the robustness of the findings, these analyses were iteratively repeated with varying seed values for random sample selection from longitudinal data pools of mild (n = 64), moderate/severe (n = 57), and non-IBD samples (n = 119). Within the HMP2 cohort, inclusion was also limited to cross-sectional samples accompanied by available calprotectin data. In response to the attenuated metagenome disease signal observed in the study cohort^73^, a targeted inflammation-specific selection approach was utilized instead of choosing the cross-sectional data via repeated random sampling. For the CD and UC sub-cohorts, the sample with maximal calprotectin value per patient was included (CD = 41, UC = 26). Conversely, for the non-IBD control group, the cross-sectional sample with the minimal calprotectin value per patient was chosen (n = 24). Statistical analyses were conducted using R software version 4.2.1 (Ubuntu 20.04.5 LTS).

### Untargeted stool metabolomics and gluconate intensity estimation

Untargeted stool metabolomics of faecal samples from the PROTECT cohort was performed using LC-MS in negative mode, and calprotectin was measured by ELISA. Briefly, hydrophilic interaction liquid chromatography (HILIC) analyses of water-soluble metabolites in the negative ionization mode were conducted using Shimadzu Nexera X2 U-HPLC (Shimadzu Corp.) coupled to a Q Exactive Plus mass spectrometer (Thermo Fisher Scientific). Metabolites were extracted from plasma or stool (30 μL) using 120 μL of 80% methanol containing inosine-15N4, thymine-d4, and glycocholate-d4 internal standards (Cambridge Isotope Laboratories). The samples were centrifuged (10 min, 9,000 × *g*, 4°C), and the supernatants were injected directly onto a 150 × 2.0 mm Luna NH2 column (Phenomenex; Torrance, CA). All masses detected in HILIC negative mode were matched via adduct subtraction and molecular formula match to compounds downloaded from the Human Metabolome Database (HMDB) on 10/10/2022. The measured m/z values were adjusted for [M-H]- adducts, and molecular formulae matching to within 5 ppm were selected as candidate identifiers. In cases where multiple molecular formulae matched the adduct-adjusted mass (as a result of multiple potential adducts), the one with a minimal ppm difference was selected. Out of 4,461 detected features (m/z, retention time pairs) a single feature 195.0512 m/z @ 4.34 min resolved to the formula C_6_H_12_O_7_ (delta ppm = 0.89), related to a group of five compounds with canonical structure O=C(O)C(O)C(O)C(O)C(O)CO, which includes L-gluconic acid (HMDB0000625).

### Statistical analyses

Statistical analyses were performed using GraphPad Prism software (GraphPad Software, Inc.). Kruskal-Wallis test and FDR method of Benjamini and Hochberg were used for multiple comparisons during CFU comparisons. Mann-Whitney U test with Welch’s correction was used for comparisons between the two groups. Spearman’s rank correlation was used to investigate the correlation between the relative abundance of Kp-2H7 and isolated strains.

## Data Availability

Genomes of the sequenced 119 strains, the 16S rRNA sequence data, RNA sequence data, and Tn-seq data will be deposited in the DNA Data Bank of Japan.
